# Dr. Brainard’s Introductory, Again

**Published:** 1856-04

**Authors:** 


### DR. BRAINARD'S INTRODUCTORY, AGAIN.

In our last, we respectfully called the attention of our brethren at Chicago, to the introductory of Prof. Brainard, in which he was pleased to speak indifferently, if not disparagingly of our institution at this place. We, of course, presumed that he was not familiar with our history, or he would not have said that "at best, we had only been partially successful." We had the charity to believe that he spoke his convictions, founded upon erroneous statements, and took occasion to point out his error, relying upon his courtesy to make the *amende* in the future. This has not been done, although we have received one number of the Medical Journal, published at that place, some time since our issue, containing this notice.

We again call the attention of our brethren to this subject, and refer them to the notice of our commencement in this number, and then, if this be not satisfactory, our forthcoming catalogue, with the names, post offices, &c., we hope will be sufficient testimony to show that our neighbor has been in error. We ask this as an act of common justice, which we would feel ourselves under obligations to render to any and all, when unintentionally we had been led, directly or indirectly, to do them an injury.

